# Long-Term Impact of Pneumococcal Conjugate Vaccines on the Burden of Pneumococcal Meningitis in Mozambique, 2013–2023

**DOI:** 10.3390/vaccines13121246

**Published:** 2025-12-15

**Authors:** Aquino Albino Nhantumbo, Goitom Weldegebriel, Linda de Gouveia, Reggis Katsande, Charlotte Elizabeth Comé, Alcides Moniz Munguambe, Vlademir Cantarelli, Cícero Dias, Rachid Muleia, Ezequias Fenias Sitoe, Eunice Veronica Zeca, Amir Seni, Ana Nicolau Tambo, Ana Cristina de Faria Neves Mussagi, Plácida Iliany Maholela, Ivano de Filippis, Eduardo Samo Gudo

**Affiliations:** 1 Instituto Nacional de Saúde, Marracuene 1120, Mozambique; charlotte.come@ins.gov.mz (C.E.C.); alcides.munguambe@ins.gov.mz (A.M.M.); rachid.muleia@ins.gov.mz (R.M.); placida.maholela@ins.gov.mz (P.I.M.); eduardo.samogudo@ins.gov.mz (E.S.G.); 2 National Institute for Quality Control in Health (INCQS), Fundação Oswaldo Cruz (FIOCRUZ), Rio de Janeiro 21040900, Brazil; ivano.defilippis@fiocruz.br; 3 World Health Organization African Regional Office, Harare 348, Zimbabwe; weldegebrielg@who.int (G.W.); katsander@who.int (R.K.); 4 National Health Laboratory Service (NHLS), National Institute for Communicable Diseases (NICD), Johannesburg 2192, South Africa; lindad@nicd.ac.za; 5 Departamento de Ciências Básicas da Saúde, Universidade Federal de Ciências de Saúde de Porto Alegre (UFCSPA), Porto Alegre 900050170, Brazil; vlademirc@ufcspa.edu.br (V.C.); cicero@ufcspa.edu.br (C.D.); 6 Nampula Central Hospital, Ministry of Health, Nampula 3100, Mozambique; ezequias5sitoe@gmail.com (E.F.S.); euniceveronicazeca@yahoo.com.br (E.V.Z.); 7 Beira Central Hospital, Ministry of Health, Sofala 2100, Mozambique; amirseni@gmail.com (A.S.); anatambo81@gmail.com (A.N.T.); 8 Maputo Central Hospital, Ministry of Health, Maputo 1100, Mozambique; hannahcristinna89@gmail.com

**Keywords:** *S. pneumoniae*, pneumococcal conjugate vaccines, vaccine coverage, children under five, Mozambique

## Abstract

Background: Mozambique introduced the 10-valent pneumococcal conjugate vaccine (PCV10) in 2013 using a three-dose primary series with no booster dose (3p+0) and later switched to the PCV13 using a schedule of two primary doses with one booster (2p+1). We aimed to describe the burden and serotype distribution of pneumococcal meningitis in children under 5 years of age in Mozambique over an eleven-year period starting with the year of PCV10 introduction, and assess the impact of the PCV vaccine and schedule changes. Methods: We analysed meningitis surveillance data in Mozambique from March 2013 through to December 2023. Cerebrospinal fluid (CSF) samples were collected from eligible children in three referral hospitals (Maputo Central Hospital [south], Beira Central Hospital [central], and Nampula Central Hospital [north]). Culture and polymerase chain reaction assay (qPCR) were performed on each sample. *S. pneumoniae*-positive samples were subsequently serotyped using multiplex assay. We estimated annual incidence rates for pneumococcal meningitis in children under 5 years old following the PCVs’ introduction (2013–2023). The impact of the product switch and schedule change from PCV10/3p+0 to PCV13/2p+1 on the burden and serotype distribution of pneumococcal meningitis was assessed. Results: Of the 4075 CSF samples tested, 7.4% (301/4075) were positive for *S. pneumoniae,* 2.5% (103/4075) for *H. influenzae*, and 1.0% (42/4075) for *N. meningitidis*. Pneumococcal meningitis incidence in children under five reduced from 44.7 cases per 100,000 in 2013 to 4.6 cases per 100,000 in 2023, an 89.7% reduction. In the PCV13/2p+1 period (2020–2023), pneumococcal meningitis incidence was 51.2% lower than the PCV10/3p+0 period (2013–2017) (IRR 0.49, 95% CI 0.4–0.6; *p* < 0.001). PCV10-serotype pneumococcal meningitis incidence among children under five decreased by 65.6% in the PCV13/2p+1 period (IRR 0.34, 95% CI 0.2–0.6; *p* < 0.001). We detected zero cases of pneumococcal meningitis due to the PCV13-serotype in 2020–2023, whereas non-PCV10/13-serotypes increased by 76% (IRR 1.76, 95% CI 1.2–2.6; *p* = 0.004). The case–fatality proportion decreased by 71.9% (95% CI 62.9–84.8%) in the PCV13/2p+1 period. Conclusions: Since the introduction of PCVs in Mozambique, the burden of pneumococcal meningitis and deaths in children under 5 years of age has substantially decreased, as well as the prevalence of PCV13-serotypes. Higher valency PCVs are needed due to the increased prevalence of non-PCV10/13-serotypes. Funding: Gavi, The Vaccine Alliance, reference number: MOZ-HSS-2-INS; WHO Reference: 2014405143-0, creation DFC to support HIB & Surveillance System.

## 1. Background

*Streptococcus pneumoniae* (pneumococcus) remains the most frequent vaccine-preventable bacterial aetiology of meningitis, pneumonia, and sepsis worldwide, with the highest burden in sub-Saharan African and south Asia [1,2,3,4]. Mortality due to invasive pneumococcal diseases (IPDs) remains high, causing an estimated 317,300 deaths in children under 5 years worldwide in 2015 [1]. Meningitis was the most severe clinical presentation of pneumococcal disease, with an estimated burden of 13 cases per 100,000 children aged 1–59 months globally in 2015 [1], and a high case fatality ratio (CFR) of up to 50% in low-income countries [1,2,3,5]. Even with proper diagnosis and treatment, about 8–15% of patients with bacterial meningitis will die, and approximately 20% of survivors may experience permanent hearing loss, brain damage, or learning disabilities [6].

The 7-valent pneumococcal conjugate vaccine (PCV7) (containing seven serotypes: 4, 6B, 9V, 14, 18C, 19F, and 23F) was the first to be licensed and used in 2000. It was replaced in 2008–2009 by two higher valency vaccines, PCV10 and PCV13, which cover 10 (PCV7 serotypes plus 1, 5, and 7F) and 13 (PCV10 serotypes plus 3, 6A, and 19A) serotypes, respectively. These vaccines are critical public health tools for reducing the burden of pneumococcal diseases and are currently used in most countries globally (mostly PCV10 and PCV13). Overall, in many countries worldwide, the implementation of the pneumococcal conjugate vaccines (PCVs) into the Expanded Program Immunization showed a remarkable reduction between 70% and 93% in the burden of pneumococcal meningitis and other invasive pneumococcal diseases (IPDs) caused by vaccine-type *S. pneumoniae* serotypes [1,5,7,8,9,10,11,12]. Moreover, evidence from a variety of settings has demonstrated that PCVs are highly effective against vaccine-type IPD and provide indirect effects, and their use has been recommended by WHO for infants worldwide [1,5,7,8,9,10,11,12].

With the support of Gavi, the Vaccine Alliance, Mozambique introduced the PCV10 into the expanded immunization program (EPI) in April 2013 using as a three-dose schedule without a booster (3p+0 schedule) administered at 6, 10, and 14 weeks of age [10]. During the first 3 years post-PCV10 introduction, a reduction in the burden of pneumococcal meningitis caused by those serotypes included in the vaccine was observed [10]. In December 2017, Mozambique switched from PCV10 to PCV13 with a phased approach starting the rollout in the Northern and Central regions of the country and was finalized nationally in May 2019, using a 3p+0 vaccination schedule; however, the use of the new vaccination schedule of two primary doses given at ages 2 and 4 months with a booster at 9 months (2p+1 schedule) began in September 2019. However, evidence reporting long-term changes on the burden of IPD and serotype dynamics over time following PCV introduction, as well as the impact of the product switch from PCV10 to PCV13 and schedule change, especially from low-income countries, remains limited. In the present study, our aims were to describe the burden and serotype distribution of pneumococcal meningitis among children under 5 years during the 11 years following PCV introduction (2013–2023), assess the impact of the product switch from PCV10 to PCV13, and schedule the change from 3p+0 to 2p+1 on the burden of pneumococcal meningitis and pneumococcal serotype distribution as well as the variables associated with pneumococcal meningits in Mozambique.

## 2. Methods

### 2.1. Study Setting and Population

Data from the routine sentinel surveillance system for pediatric bacterial meningitis in Mozambique were used in this study. The Instituto Nacional de Saúde (INS) of Mozambique has been conducting ongoing hospital-based surveillance for vaccine-preventable bacterial meningitis since 2013 at the three largest referral hospitals in the Mozambique-Maputo Central Hospital (MCH), Beira Central Hospital (BCH), and Nampula Central Hospital (NCH) located in the southern, central, and northern regions of the country, respectively [13]. The catchment population of a specific administrative area within each province was based on the 2007 census (population from 2013 to 2016) and 2017 census (population from 2017 to 2023). However, the <5 year old population (660,231 in 2013–2017; 553,793 in 2020–2023) is covered by MCH; (311,862 in 2013–2017; 510,154 in 2020–2023) by BCH; and (480,046 in 2013–2017; 671,322 in 2020–2023) by NCH (Supplementary Table S1) [14]. The population covered by each hospital for each year (Supplementary Table S1) was used as a denominator to estimate the annual pneumococcal meningitis incidence. All of these hospitals offer several specialized services for all age groups and also receive all cases of meningitis notified in hospitals within the catchment area of each hospital due to their capacity to manage meningitis cases.

The study was divided into three periods: the PCV10/3p+0 period (2013–2017), transition period (2018–2019), and the PCV13/2p+1 period (2020–2023) to assess the impact of the product switch from PCV10 to PCV13 and schedule a change from 3p+0 to 2p+1 on the burden of pneumococcal meningitis and pneumococcal serotype distribution in Mozambique.

### 2.2. Case Definition and Enrollment

As part of the surveillance system for paediatric meningitis in Mozambique, children under 5 years old, hospitalized at each of these three sentinel sites who met WHO case definitions for suspected meningitis [15], were enrolled in this study. Upon admission, patients with suspected meningitis underwent lumbar puncture by trained physicians followed by the completion of a standardized case report form (CRF) [13].

The vaccination status was defined as unvaccinated (children that received zero doses of PCV10/13 vaccines), partially vaccinated (children that received one or two doses of PCV10/13), or fully vaccinated (children that received three doses of PCV10/13 vaccines). Vaccination status was confirmed from the child’s health card and the health facility registries or the parent or guardian reported no vaccine doses since birth.

### 2.3. Sample and Data Collection

Cerebrospinal fluid (CSF) specimens, patient basic demographic and clincial data, as well as HIV status ascertainment that is routinely tested for all children at the health facilities were collected prospectively from all suspected meningitis cases at each sentinel site using a standard case report form (CRF) previously described [13]. CSF specimens were immediately sent to their respective clinical microbiology laboratory for routine diagnostic tests [13].

### 2.4. Laboratory Methods

***S. pneumoniae* identification:** As part of the sentinel surveillance system for meningitis, all collected CSF specimens from enrolled children were first processed at the local laboratories for microscopy (cell count), glucose and protein level, and Gram stain, and bacterial culture conducted using 5% sheep blood and chocolate agar plates (MAST, Merseyside, UK) [10,13]. Pneumococci were identified based on morphological features in a Gram stain, optochin susceptibility (OXOID-DD1 OPTOCHIN, Basingstoke, UK), and bile solubility test (BD–BBL Desoxycholate Reagent Droppers, Becton Dickinson and Company, Franklin Lakes, NJ, USA) [13]. Cultured pneumococcal isolates (stored in skim milk, tryptone, glucose, and glycerin medium) and any residual CSF were frozen at minus seventy degrees Celsius (−70 °C) once routine diagnostic tests were completed and shipped on dry ice to the National Reference Microbiology Laboratory (NMRL) at the INS of Mozambique. The triplex real-time PCR assay (RT-PCR) [16] was used for the detection of *H. influenzae*, *N. meningitidis*, and *S. pneumoniae* from all samples with a cycle threshold (ct) value ≤ 35 considered positive.

**Determination of Pneumococcal Serotypes:** All pneumococcal isolates and *S. pneumoniae* PCR-positive CSF samples from 2013 were serotyped at Universidade Federal de Ciências de Saúde de Porto Alegre (UFCSPA), Brazil, using the sequential multiplex conventional PCR (SM-PCR), covering 40 serotypes [17]. From 2016 to 2020, pneumococcal isolates and *S. pneumoniae*-positive CSF samples were serotyped at the National Institute for Communicable Diseases (NICDs) of the National Health Laboratory Service (NHLS) in South Africa by performing 8 sequential multiplex quantitative PCR methods described by Pimenta et al. (2013) [17], adapted for the regional algorithm, and covering the 38 most common serotypes involved in the aetiology of IPD in Africa [17]. From 2020 onwards, *S. pneumoniae*-positive CSF samples were serotyped at the Instituto Nacional de Saúde in Mozambique by using real-time PCR assays in 12 quadriplex reactions, covering 48 serotypes including serotypes 22F, 22A, 6AB, 6AB, 6BD, 31, 7C/7B1, 34, 38, 28A/28F, 37, 11B/11C, 10F, and 35A not covered by previous methods [18].

Confirmed cases of pneumococcal meningitis were defined as the presence of *S. pneumoniae,* identified either by culture or detected in CSF using multiplex qPCR [19].

Pneumococcal meningitis cases were classified into categories as follows: PCV10 serotypes (1, 4, 5, 6B, 7F, 9V, 14, 18C, 19F, or 23F), PCV13 serotypes (PCV10 serotypes plus 3, 6A, and 19A), PCV15 serotypes (PCV13 serotypes plus 22F and 33F), PCV20 serotypes (PCV15 serotypes plus 8, 10A, 11A, 12F, and 15B), and non-PCV10/13 serotypes (all other serotypes including non-typeable pneumococci and PCR negative for the 40 serotypes included in the Multiplex real-time PCR). All samples with low deoxyribonucleic acid (DNA) concentration (more than 35 Ct values) and insufficient CSF were classified as missing cases.

### 2.5. Statistical Analysis

Statistical analyses were conducted using the R statistical software version 4.1.1 (Vienna, Austria). Annual incidence rates were calculated as the number of cases per age group divided by the risk age-specific population multiplied by 100,000 with exact Poisson confidence intervals. The denominator used to calculate the incidence was the catchment area population of each sentinel site for each year (Suplementary Table S1), estimated from the 2007 census (population from 2013 to 2016) and 2017 census (population from 2017 to 2023) census stratified by age (0–23 months and 24–59 months) [14]. To assess the impact of the product switch from PCV10 to PCV13 and the schedule change from 3p+0 to 2p+1, incidence rate ratios were calculated by dividing the PCV13/2p+1 period (2020–2023) incidence rates by the baseline incidence rates (PCV10/3p+0 period (2013–2017)) with exact Poisson confidence intervals. We also assessed the annual incidence changes on the PCV10/13 and non-PCV10/13 serotype pneumococcal meningitis per 100,000 children under 5 years old between March 2013 and December 2023. Mortality was determined as the number of deaths due to pneumococcal meningitis divided by the total population at risk (children under 5 years). The case fatality rate was determinated as the proportion of people who have been diagnosed with pneumococcal meningitis and end up dying due to this disease. The multivariate logistic regression model was used to determine the variables associated with pneumococcal meningits. In this model, variables were included if their *p*-value in the bivariate logistic regression was less than 25%. Statistical significance in the multivariate logistic regression was assessed at the 5% significance level. For the multivariate logistic regression, the adjusted Odds Ratio (aOR) and 95% confidence interval [95% CI] are reported.

### 2.6. Ethics Statement

Ethical approval for this study was given by the Mozambican National Bioethics Committee Ref #: 180/CNBS/20/IRB00002657. Verbal consent was obtained from all the participating children’s parents during routine medical care for paediatric bacterial meningitis in Mozambique [13].

## 3. Results

### 3.1. General Characteristics of Study Participants

**Figure 1:**
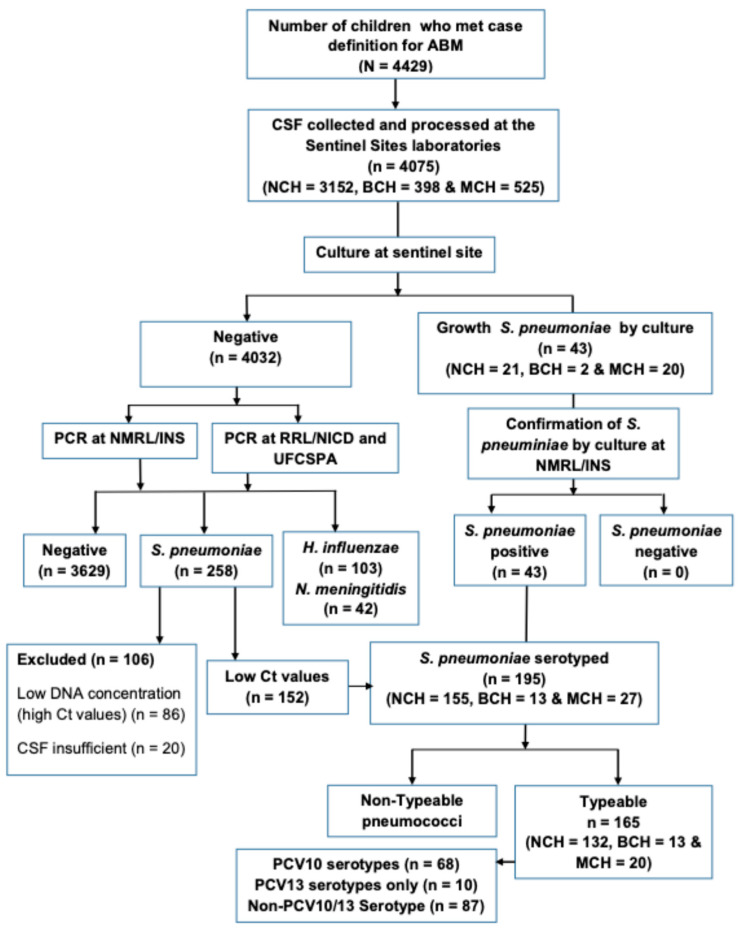
Study profile during the observation period March 2013 to 31 December 2023. The flow chart depicts the total number of suspected bacterial meningitis among children under 5 years old and number of CSF samples collected and tested between March 2013 and December 2023. Abbreviations. **ABM:** Acute bacterial meningitis; **BCH:** Beira Central Hospital; **CSF:** Cerebrospinal fluid**; Ct:** Cycle threshold**; DNA:** Deoxyribonucleic acid; **INS:** Instituto Nacional de Saúde; **MCH:** Maputo Central Hospital**; NCH:** Nampula Central Hospital; **NICD:** National Institute for Communicable Diseases; **NMRL:** National Reference Microbiology Laboratory; **NTS:** non-typable specimen; **PCR:** polymerase chain reaction; **PCV13:** 13-valent pneumococcal conjugate vaccine; **RRL:** Regional Reference Laboratory; **UFCSPA:** Universidade Federal de Ciências de Saúde de Porto Alegre.

Between March 2013 and December 2023, of a total of 4429 children <5 years with suspected meningitis across the three largest hospitals included in the surveillance system, 4075 (92.0%) cases had CSF specimen collected (Figure 1). The median age was 11 months (Interquartile Range (IQR) 4–25 months), 55.8% (2273/4075) were male, and more than half of the children enrolled (66.6%, 2712/4075) were aged less than 24 months (Table 1). Most of the study participants were from NCH (77.3%), 9.8% from BCH, and 12.9% from MCH (Figure 1, Table 1).

### 3.2. Prevalence and Incidence Trends of Pneumococcal Meningitis Among Children with Suspected Meningitis in the Period Between 2013 and 2023

**Figure 2:**
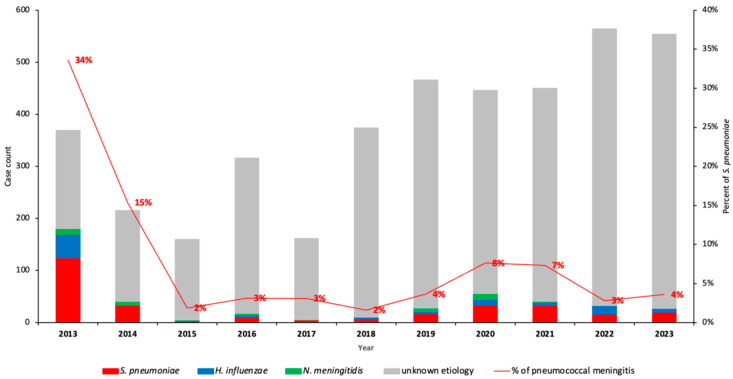
**Detection of *S. pneumoniae, N. meningitidis,* and *H. influenzae* in CSF samples by PCR from 2013–2023 in Mozambique**. This figure depicts the annual variation of the number of bacterial meningitis and relative frequency of *S. pneumoniae* causing pneumococcal meningitis and also the variation in the number of CSF samples collected from children <5 years. Frequency of *S. pneumoniae* was determined using PCR. Abbreviations: **CSF**, cerebrospinal fluid; **PCR**, polymerase chain reaction.

During the 11-year surveillance period (2013–2023), we identified 7.4% (301/4075) *S. pneumoniae* among children under 5 years old with suspected meningitis, respectively (Figure 1, Supplementary Dataset S1). Overall, the proportion of all confirmed pneumococcal meningitis cases decreased significantly from 34.0% in 2013 to 4.0% in 2023 (*p* < 0.001). This represents a relative decrease of 83.9% in pneumococcal meningitis (*p* < 0.001) when compared to 2013 (Figure 2).

**Figure 3:**
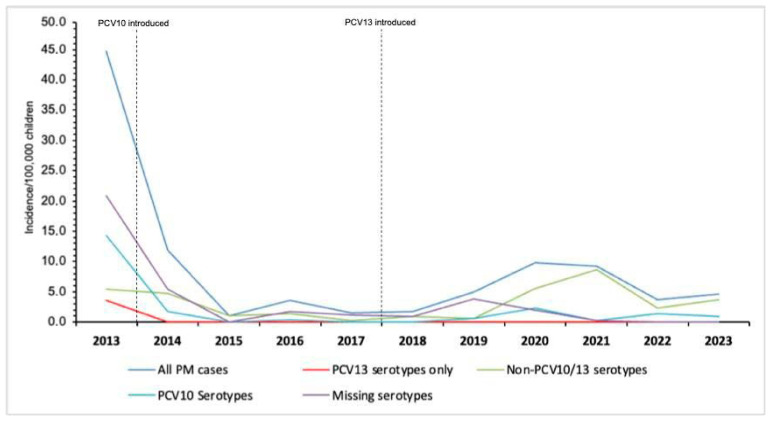
Incidence of overall PCV10/13 serotypes and non-PCV10/13 serotypes pneumococcal meningitis in Mozambique, 2013–2023. This figure describes the annual incidence of pneumococcal meningitis among children following the introduction of pneumococcal conjugate vaccines, using routine data of the Meningitis Surveillance System in Mozambique, 2013–2023. Abbreviations: PCV, pneumococcal conjute vaccine; PM, pneumococcal meningitis.

The annual incidence of overall pneumococcal meningitis cases among children under 5 years old reduced significantly from 44.7 cases per 100,000 in 2013 (the year of PCV10 introdution) to 4.6 cases per 100,000 in 2023, equivalent to a 89.7% reduction (*p* < 0.001) (Figure 3). Within the same period (2013–2023), the incidence of pneumococcal meningitis caused by PCV10 serotypes decreased from 14.3 to 0.9 cases per 100,000 in 2013 and 2023, respectively (93.7% decrease) (Figure 3, Supplementary Dataset S1). Pneumococcal meningitis incidence due to PCV13 only (serotypes 3, 6A, and 19A) was 3.6 cases per 100,000, with no cases detected from 2014 onwords. However, a six-fold increase of non-PCV10/13 pneumococcal serotype meningitis among children under 5 years old was evident from 0.6 to 3.7 cases per 100,000 between 2019 and 2021, respectively (Figure 3).

### 3.3. Incidence of Pneumococcal Meningitis During the PCV10/3+0 Period (2013–2017) and PCV13/2+1 Period (2020–2023)

Overall, peumococcal meningitis incidence was lower during the PCV13/2p+1 period than the PCV10/3p+0 period (5.9 vs. 12.1 cases per 100,000; IRR 0.49, 95% CI 0.4–0.6; *p* < 0.001; Table 2, Figure 3). Among children younger than two years, pneumococcal meningitis incidence also decreased significantly from 15.8 cases per 100,000 during the PCV10/3p+0 period (*p* < 0.001) to 7.8 cases per 100,000 in the PCV13 (2p+1) period, equivalent to a 50.6% reduction (95% CI 60.2–88.6; *p* < 0.001) (Table 2, Figure 3). In children above 2 years of age, pneumococcal meningitis in the PCV13/2p+1 period was lower compared with the PCV10/3p+0 period (3.2 vs. 6.8 cases per 100,000; IRR 0.56, 95% CI 0.4–0.6; *p* < 0.001) (Table 2, Figure 3). Within the same period, the incidence of pneumococcal meningitis was significantly lower among study sites [MCH: IRR 0.05, 0.0–0.2; *p* < 0.001] and [NCH: IRR 0.53, 0.4–0.7; *p* < 0.001]) in 2020–2023 compared with 2013–2017 and in both HIV-infected and non-HIV infected children (Supplementary Table S2). By contrast, no significant reduction was observed in Beira Central Hospital in 2020–2023 (IRR 1.50, 95% CI 0.5–4.2; *p >* 0.05) (Supplementary Table S2).

### 3.4. Trends on Serotype Distribution and Vaccine Formulation Coverage

**Figure 4:**
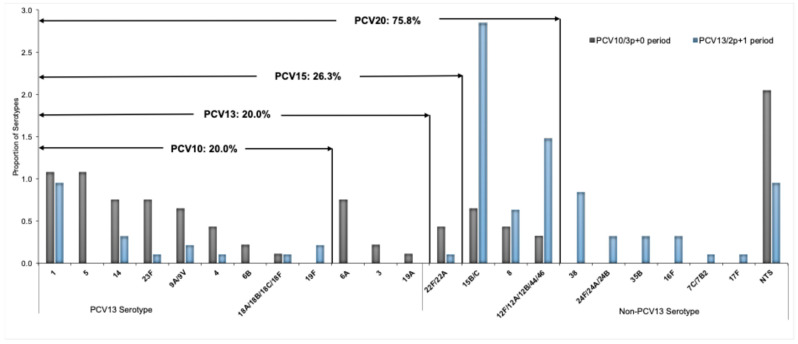
Distribution and coverage of *S. pneumoniae* serotypes by PCV10, PCV13, PCV15, and PCV20, between PCV10/3p+0 period (2013–2017) and PCV13/2p+1 period (2020–2023). Each bar represents the proportion of isolates with each serotype of *S. pneumoniae.* The value in the arrows above the bars depicts the coverage of the *S. pneumoniae* serotype by PCV10, PCV13, PCV15, and PCV20, respectively. NTS, specimen negative for 40 serotypes by Multiplex real-time PCR. Abbreviations: **NTS**, non-typable specimen; **PCR**, polymerase chain reaction; **PCV**, pneumococcal conjute vaccine.

Serotyping of *S*. *pneumoniae* was performed in a total of 195 (64.8%) of 301 samples, comprising the 43 isolates of *S*. *pneumoniae* identified by culture and 152 PCR-positive CSF specimens. Of the 195 *S. pneumoniae* serotyped, 47.7% (93/195) were from the PCV10/3p+0 period (2013–2017), 3.6% (7/195) were from the transition period (2018–2019), and 48.7% (95/195) were from the PCV13/2p+1 period (2020–2023) (Table 3). The incidence of pneumococcal meningitis due to PCV10 serotypes among children under 5 years old in the PCV10/3p+0 period was 3.2 per 100,000, decreasing to 1.1 per 100,000 in the PCV13/2p+1 period (IRR 0.34, 95% CI 0.2–0.6; *p* < 0.001). Pneumococcal meningitis incidence due to PCV13-only serotypes (serotypes 3, 6A, and 19A) in the PCV10/3p+0 period was 0.7 cases per 100,000, and no cases were detected during the PCV13/2p+1 period (Table 2, Figure 3). By contrast, non-PCV10/13 pneumococcal meningitis incidence was significantly higher (4.4, 95% CI 3.4–5.4; *p* < 0.001) in 2022–2023 compared with the 2013–2017 period across all ages, except in children aged 24–59 months, for whom it was lower (Table 2, Figure 3). The most predominant identified serotypes during the PCV10/3p+0 period (2013–2017) were 1 (10.8%, 10/93), 5 (10.8%, 10/93), 14 (7.5%, 7/93), 23F (7.5%, 7/93), and 6A (7.5%, 7/93) (Figure 4, Table 3). In the PCV13/2p+1 period (2020–2023), the most frequently identified serotypes were 15B/C (28.4%, 27/95), 12F/12A/12B/44/46 (14.7%, 14/95), 1 (9.5%, 9/95), 38 (8.4%, 8/95), and 8 (6.3%, 6/95) (Figure 4, Table 3). Moreover, the rate of vaccine coverage against the serotypes of *S*. *pneumoniae* causing paediatric meningitis in Mozambique during the PCV13 (2p+1) period (2020–2023) was 20.0% (19/95), 20.0% (19/95), 26.3% (25/95), and 75.8% (72/95) for PCV-10, PCV-13, PCV-15, and PCV-20, respectively (Figure 4).

In the annual analyses of serotype-specific changes after the PCVs’ introduction in Mozambique, the reduction in the most predominant PCV10 and PCV13 serotypes was evident from 2013 onwards (Supplementary Figure S1A). However, the non-PCV10/13 serotype decreased from 2013 to 2018, increased after the PCVs’ introduction, and became the most common circulating serotype (Supplementary Figure S1B).

### 3.5. Mortality and Case Fatality Ratio

From 2013 to 2023, pneumococcal meningitis mortality in children younger than 5 years decreased significantly in Mozambique from 22 to 0.4 deaths per 100,000 (*p* < 0.001) (Supplementary Table S3). On the other hand, the case fatality ratio (CFR) among children under 5 years old suffering from pneumococcal meningitis reduced significantly from 18.3% (32/175 [95% CI: 12.9–24.8%]) in the PCV10/3p+0 vaccine period (2013–2022) to 8.7% (9/103 [95% CI: 6.75–10.93%]) in the PCV13 vaccine period (2020–2023) (*p* = 0.007) (Supplementary Table S3).

### 3.6. Predicting Variables Associated with Pneumococcal Meningitis Infection Among Children Under 5 Years Old

Table 4 represents the analysis of predicting variables associated with pneumococcal meningitis infection among children under 5 years in Mozambique. In the multivariable analysis, we found that pneumococcal meningitis was associated with ages below 23 months, using the age group of 24–59 months as a reference (adjusted OR (aOR) = 1.6; 95% CI 1.19–2.21; *p* = 0.002), HIV-positive status (aOR = 23.0, 95% CI 10.51–50.35; *p* < 0.001), and vaccination status (unvaccinated children) (aOR = 29.41, 95% CI 17.29–50.02; *p* < 0.001). No association was found between pneumococcal meningitis and geographical regions, although there was a slight trend towards a higher incidence of pneumococcal meningitis in Nampula, in the northern of Mozambique.

## 4. Discussion

Mozambique introduced the PCV10 vaccine formulation in 2013 and then switched to PCV13 in December 2017, which was finalized nationally in May 2019. In this study, we reported for the first time the long-term changes on the incidence of pneumococcal meningitis in children under 5 years old and the impact that the product switch from PCV10 to PCV13 and the schedule change from 3p+0 (2013–2017) to 2p+1 (2020–2023) had on the burden of IPD and pneumococcal serotype distribution in Mozambique. As expected, the data from our study highlighted a large reduction equivalent to 89.7% in the incidence of overall pneumococcal meningitis and a 93.7% reduction in PCV10 serotype pneumococcal meningitis following the introduction of PCV vaccines in the EPI program in Mozambique between 2013 and 2023. In addition, administrative data on vaccine coverage obtained from the EPI program in Mozambique show that during this period, the coverage of PCV10 and PCV13 vaccines was greater than 90% (Supplementary Figure S2), which gives remarkable consistency to the findings of our study. The findings from this study are in line with those previously reported in many African countries [20,21,22,23], Europe [24,25], Asian countries [26,27], America (Latin America) [28,29], and in the USA [30] where PCV vaccines were introduced. In these countries, there was a rapid reduction in invasive pneumococcal disease, including pneumococcal meningitis between 70% and 93%, following the implementation of pneumococcal conjugate vaccines.

In this study, we note an important remarkable decrease in vaccine-type pneumococcal meningits observed among children under five during the PCV13/2p+1 period (2020–2023) that had been the most prevalent in the country since the introduction of the PCV10 formulation vaccine. Our results are consistent with other previously reported findings in many countries, where PCV10 or PCV13 were also used [25,26,27,28,29,30,31,32].

There was a noticeable annual serotype-specific change after the pneumococcal conjugate vaccines’ (PCV10 and PCV13) introduction in Mozambique. The most common PCV10 (serotypes 1, 3, 5, 6B, 19A, 23F, and 14) and PCV13 serotypes (serotypes 3, 6A, and 19A) decreased significantly from 2013 onwards. No serotypes 3, 4, 5, 6B, 6A, 9V/9A, 14, 19A, and 23F were detected 11 years after pneumococcal conjugate vaccines were implemented in the country (Supplementary Figure S2A, Supplementary Dataset S1). These findings are in line with previous observations and underline the impact of vaccination strategies to strengthen herd protection in children under 5 years old [20,21,22,23]. For serotype 1, there is strong evidence of 2p+1 impact on disease [33]. However, our findings show that serotype 1 declined and disappeared for a few years, and then once the schedule changed to 2 + 1, it reappeared, albeit in low proportion. The children with pneumococcal meningitis caused by serotype 1 during the PCV13/2p+1 period were unvaccinated (Supplementary Dataset S1).

Another remarkable finding in our study was the significant increase in non-PCV10/13 (NVT) serotypes observed during the PCV13/2p+1 period (2020–2023). The main NVTs include 15B/C, 12F/12A/12B/44/468, 22F/22A, 38, 8, 35B, 24F/24A/24B, and 16F, which was common among 80.0% of the children under five years old with confirmed pneumococcal meningitis (Supplementary Dataset S1). The increase in NVTs may be related to the serotype replacement phenomenon in Mozambique. These findings are similar to those previously reported in other countries worldwide, which sustained the use of the PCV10 or PCV13 formulation vaccines in the paediatric National Immunization Program [32,33,34,35]. These countries have witnessed an increased incidence of NVT serotypes such as serotypes 8, 12F, 15B, 15A, 22F, 33, as a result of serotype replacement among invasive pneumococcal disease cases in children [32,33,34,35]. The serotype replacement phenomenon post-PCVs’ introduction has been repeatedly reported worldwide because of the emergence of non-vaccine types [32,33,34,35,36,37,38,39]. Based on changes in the incidence rates of PCV serotypes, we suggest the development of formulations which include some of the evolving, as this may help further reduce the current pneumococcal disease burden across all ages in Mozambique.

The case fatality ratio among children under 5 years old with confirmed pneumococcal meningitis reduced by 71.9% during the PCV13/2p+1 period (2020–2023) in the country. The reduction in the country’s burden and mortality due to pneumococcal meningitis could be attributed to successful vaccination rollouts over the past 11 years, using highly effective pneumococcal conjugate vaccines as well as the reduction in most PCV10 and PCV13 serotypes. These findings are in line with those previously reported in many countries [1,40]. Reducing cases and deaths due to vaccine-preventable bacterial meningitis in 50% and 70%, respectively, is one of the main goals of the WHO global roadmap; thus, continued vaccination against invasive pneumococcal disease is essential [41]. We recognize that various ongoing public health interventions such as strengthened health systems, improved case management and improved nutrition, the administration of vitamin A supplementation, and the availability of quality diagnosis, treatment, and care services for maternal, newborn, and child health prevention of mother-to-child transmission of HIV are contributing to the decrease in case fatality (Unpublished data of Ministry of Health Mozambique). Additionally, the reduction in the country’s burden due to pneumococcal meningitis could be attributed to the public health and social measures implemented due to the COVID-19 pandemic. A substantial reduction in invasive pneumococcal disease has been reported globally during the COVID-19 pandemic [42,43,44].

An assessment was conducted of the predicting variables associated with pneumococcal meningitis among children under 5 years old. Age under 2 years, HIV-positive status, and non-vaccination with PCV were strongly associated with pneumococcal meningitis, which is similar to the findings from other authors [42,43]. HIV-infected children and adults remain at high risk of IPD and death, even with antiretroviral therapy (ART) [45,46,47,48]. Thus, pneumococcal conjugate vaccines (PCVs) have been shown to be safe and highly effective in preventing IPD in both children with and those without HIV infection worldwide [45,46,47,48].

We would like to recognize some limitations of our study, which may have influenced the interpretation of the results: First, our study was conducted in three of the the largest hospitals in Mozambique, which may not be representative of the overall population, but represents as a reference hospital for each geographic region of the country. Second, the administrative coverage data were calculated by dividing the reported number of administered doses by population estimates for children aged 2–11 months projected from the 2007 and 2017 national census, although sometimes the vaccines are given to children outside the eligible age range and population denominators are overestimated; for this reason, administrative coverage can exceed 100%. Third, the interruption in vaccine-preventable disease surveillance activities due to the COVID-19 pandemic may have hindered the detection and timely notification of meningitis cases, resulting in delays in specimen transportation and laboratory confirmation of suspected cases. Last, our molecular multiplex PCR serotyping assay targets only 40 of the most common serotypes/serogroups, and we were thus unable to serotype some isolates. In addition, the proportion of positive residual CSF samples not serotyped varied by year due to insuficient CSF or low DNA concentration. However, the number of positive CSF samples for *S. pneumoniae* that were not serotyped was very low during the PCV13/2p+1 period.

## 5. Conclusions

Our study provides long-term evidence on the impact of pneumococcal vaccination on pneumococcal meningitis in Mozambique, showing a rapid and consistent decrease in the incidence and case fatality rates of pneumococcal meningitis in children under 5 years old, as well as a decrease in vaccine-type *S. pneumoniae* serotypes during the PCV13/2p+1 period in Mozambique. Next-generation PCVs with a wider serotype coverage (PCV20) to address the evolving NVTs are needed. Ongoing surveillance efforts are essential to keep monitoring these changes, including serotype replacement, in order to guide further policy decisions.

## Figures and Tables

**Table 1:** Baseline demographic characteristics of patients with pneumococcal meningitis and vaccination status by period.

Characteristics	Total	PCV10/3p+0 Period (2013–2017)		Transition Period (2013–2017)		PCV13/2p+1 Period (2020–2023)	
		ABM	*S. pneumoniae* Positive	ABM	*S. pneumoniae* Positive	ABM	*S. pneumoniae* Positive
**Total**	4075	1221	175 (14.3%)	840	23 (2.7%)	2014	103 (5.1%)
**Age in months, median (IQR)**	11 (4–25)	14 (6–36)	9 (5–23)	12 (5-26)	11 (6–36)	9 (4–24)	11 (5–24)
**Age group**							
0–23 months	2712 (66.6%)	734 (60.1%)	134 (76.6%)	302 (36.0%)	14 (60.9%)	1440 (71.5%)	73 (70.9%)
24–59 months	1363 (33.4%)	487 (39.9%)	41 (23.4%)	538 (64.0%)	9 (39.1%)	574 (28.5%)	30 (29.1%)
**Gender**							
Male	2273 (55.8%)	683 (55.9%)	98 (56.0%)	469 (55.8%)	14 (60.9%)	1121 (55.7%)	57 (55.3%)
Female	1802 (44.2%)	538 (44.1%)	77 (44.0%)	371 (44.2%)	9 (39.1%)	893 (44.3%)	46 (44.7%)
**Study sites**							
NCH	3152 (77.3%)	694 (56.8%)	121 (69.1%)	736 (87.6%)	18 (78.3%)	1722 (85.5%)	89 (86.4%)
BCH	398 (9.8%)	191 (15.6%)	5 (2.9%)	41 (4.9%)	5 (21.7%)	166 (8.2%)	12 (11.7%)
MCH	525 (12.9%)	336 (27.5%)	49 (28.0%)	63 (7.5%)	0 (0.0%)	126 (6.3%)	2 (1.9%)
**HIV status**							
Positive	47 (1.2%)	18 (1.5%)	16 (9.1%)	9 (1.1%)	1 (4.3%)	20 (1.0%)	11 (10.7%)
Negative	4028 (98.8%)	1203 (98.5%)	159 (90.9%)	831 (98.9%)	22 (95.7%)	1994 (99.0%)	92 (89.3%)
**PCV doses**							
0	739 (18.1%)	452 (37.0%)	155 (88.6%)	64 (7.6%)	15 (65.2%)	223 (11.1%)	69 (67.0%)
1 or 2	857 (21.0%)	84 (6.9%)	2 (1.1%)	118 (14.0%)	3 (13.0%)	655 (32.5%)	12 (11.7%)
3	2479 (60.8%)	685 (56.1%)	18 (10.3%)	658 (78.3%)	5 (21.7%)	1136 (56.4%)	22 (21.3%)
**Case Fatality Rate (%)**	114 (2.8%)	56 (4.6%)	32 (18.3%)	17 (2.0%)	0 (0.0%)	41 (2.0%)	9 (8.8%)

Note: **ABM:** Acute Bacterial Meningitis; **MCH:** Maputo Central Hospital; **BCH:** Beira Central Hospital; **NCH:** Nampula Central Hospital; **HIV:** Human Immunodeficiency Virus; **PCV:** pneumococcal conjugate vaccine.

**Table 2:** Comparison of pneumococcal meningitis incidence between PCV10/3p+0 period (2013–2017) and PCV13/2p+1 period (2020–2023).

Characteristic	PCV10/3p+0 Period (2013–2017)			PCV13/2p+1 Period (2020–2023)			PCV13 Period vs. PCV10 Period	
	Cases (n)	Children at Risk	Incidence per 100,000 (95% CI)	Cases (n)	Children at Risk	Incidence per 100,000 (95% CI)	Incidence Rate Ratio (95% CI)	*p*-Value
**Overal *S. pneumoniae* by age**								
**All ages**	175	1,452,139	12.1 (10.3–13.8)	103	1,735,269	5.9 (4.8–7.1)	0.49 (0.4–0.6)	<0.001
0–23 months	134	849,239	15.8 (13.0–18.5)	73	937,445	7.8 (4.3–8.9)	0.49 (0.3–0.5)	<0.001
24–59 months	41	602,900	6.8 (4.7–8.9)	30	797,823	3.8 (2.1–4.3)	0.56 (0.3–0.8)	0.012
**PCV10 ST by ages**								
**All ages**	47	1,452,139	3.2 (2.3–4.2)	19	1,735,269	1.1 (0.6–1.6)	0.34 (0.2–0.6)	<0.001
0–23 months	32	849,239	3.8 (2.5–5.1)	10	937,445	1.1 (0.3–1.2)	0.29 (0.1–0.4)	<0.001
24–59 months	15	602,900	2.5 (1.2–3.7)	9	797,823	1.1 (0.3–1.6)	0.44 (0.2–0.9)	0.05
**PCV13 ST by age**								
**All ages**	10	1,452,139	0.7 (0.3–1.1)	0	1,735,269	0.0	---	---
0–23 months	8	849,239	0.9 (0.3–1.6)	0	937,445	0.0	---	---
24–59 months	2	602,900	0.3 (0.1–0.8)	0	797,823	0.0	---	---
**Non-PCV10/13**								
**All ages**	36	1,452,139	2.5 (1.7–3.3)	76	1,735,269	4.4 (3.4–5.5)	1.76 (1.2–2.6)	0.004
0–23 months	27	849,239	3.2 (2.0–4.4)	56	937,445	6.0 (3.2–5.4)	1.88 (0.9–2.1)	<0.001
24–59 months	9	602,900	1.5 (0.5–2.5)	20	797,823	2.5 (1.2–3.1)	1.67 (0.6–3.1)	0.192

**Table 3:** Distribution of pneumococcal serotypes by period (2013–2017; 2018–2019; and 2020–2023).

PCR Results	PCV10 (3p+0) Period (2013–2017)	Transition Period (2018–2019)	PCV13 (2p+1) Period (2020 –2023)	Total
**PCV10 serotypes**				
1	10 (10.8%)	1 (14.3%)	9 (9.5%)	20 (10.3%)
5	10 (10.8%)	0 (0.0%)	0 (0.0%)	10 (5.1%)
14	7 (7.5%)	0 (0.0%)	3 (3.2%)	10 (5.1%)
23F	7 (7.5%)	0 (0.0%)	1 (1.1%)	8 (4.1%)
9V/9A	6 (6.5%)	0 (0.0%)	2 (2.1%)	8 (4.1%)
4	4 (4.3%)	0 (0.0%)	1 (1.1%)	5 (2.6%)
6B	2 (2.2%)	0 (0.0%)	0 (0.0%)	2 (1.0%)
18A/18B/18C/18F	1 (1.1%)	1 (14.3%)	1 (1.1%)	3 (1.5%)
19F	0 (0.0%)	0 (0.0%)	2 (2.1%)	2 (1.0%)
7F	0 (0.0%)	0 (0.0%)	0 (0.0%)	0 (0.0%)
**PCV13 serotypes**				
6A	7 (7.5%)	0 (0.0%)	0 (0.0%)	7 (3.6%)
3	2 (2.2%)	0 (0.0%)	0 (0.0%)	2 (1.0%)
19A	1 (1.1%)	0 (0.0%)	0 (0.0%)	1 (0.5%)
**Non PCV10/13 serotypes**				
15B/C	6 (6.5%)	0 (0.0%)	27 (28.4%)	33 (16.9%)
8	4 (4.3%)	2 (28.6%)	6 (6.3%)	12 (6.2%)
22F/22A	4 (4.3%)	1 (14.3%)	1 (1.1%)	6 (3.1%)
12	3 (3.2%)	0 (0.0%)	14 (14.7%)	17 (8.7%)
38	0 (0.0%)	0 (0.0%)	8 (8.4%)	8 (4.1%)
16F	0 (0.0%)	0 (0.0%)	3 (3.2%)	3 (1.5%)
24F/24A/24B	0 (0.0%)	0 (0.0%)	3 (3.2%)	3 (1.5%)
35B	0 (0.0%)	0 (0.0%)	3 (3.2%)	3 (1.5%)
7C/7B2	0 (0.0%)	0 (0.0%)	1 (1.1%)	1 (0.5%)
17F	0 (0.0%)	0 (0.0%)	1 (1.1%)	1 (0.5%)
NTP	19 (20.4%)	2 (28.6%)	9 (9.5%)	30 (15.4%)
Total serotypes	93 (53.1)	7 (30.4%)	95 (92.2%)	195 (64.8%)
Missing serotypes	82 (46.9%)	16 (69.6%)	8 (7.8%)	106 (35.2%)
Total	175	23	103	301

Note: **NTP**: non-typeable pneumococci; **PCV**: pneumococcal conjugate vaccine.

**Table 4:** Multivariate analyses of variables associated with pneumococcal meningitis infection in children under 5 years of age in Mozambique.

Variable	PM (n = 301)	CSF Negative (n = 3774)	Unadjusted OR [95% CI]	*p*-Value	Adjusted OR [95% CI]	*p*-Value
**Age (months)**						
0–23	221 (73.4%)	2491 (66.0%)	1.42 (1.09–1.85)	0.009	1.62 (1.19–2.21)	0.002
24–59 *	80 (26.6%)	1283 (34.0%)	1.00		1.00	
**Gender**						
Female *	132 (43.9%)	1670 (44.3%)	1.00		1.00	
Male	169(56.1%)	2104 (55.7%)	1.02 (0.80–1.29)	0.894	1.20 (0.92–1.88)	0.183
**Sites**						
BCH *	22 (7.3%)	376 (10.0%)	1000		1000	
MCH	51 (16.9%)	474 (12.6%)	1.84 (1.10–3.09)	0.021	1.34 (0.74–2.41)	0.333
NCH	228 (75.7%)	2924 (77.5%)	1.33 (0.85–2.09)	0.212	1.30 (0.78–2.18)	0.310
**HIV Status**						
Negative *	273 (90.7%)	3755 (99.5%)	1.00		1000	
Positive	28 (9.3%)	19 (0.5%)	20.27 (11.18–36.76)	<0.001	23.00 (10.51–50.35)	<0.001
**PCV-13 doses**						
1 or 2 *	17 (5.6)%	840 (22.3%)	1000		1000	
0	239 (79.4%)	500 (13.2%)	23.62 (14.26–39.10)	<0.001	29.41 (17.29–50.02)	<0.001
3	45 (15.0%)	2434 (64.4%)	0.91 (0.52–1.60)	0.753	1.20 (0.67–2.15)	0.551

Note: **CI**: confidence interval; **BCH:** Beira Central Hospital; **MCH:** Maputo Central Hospital; **NCH:** Nampula Central Hospital; **HIV:** Human Immunodeficiency Virus; **OR:** Odds Ratio; **PCV:** pneumococcal conjugate vaccine; **PM:** pneumococcal meningitis; * Reference Category.

## Data Availability

All relevant data are presented in the paper.

## Supplementary Materials

The following supporting information can be downloaded at: https://www.mdpi.com/article/10.3390/vaccines13121246/s1.

## Abbreviations

**ABM:** acute bacterial meningitis; **CSF:** cerebrospinal fluid; **Ct:** Cycle threshold; **DNA:** deoxyribonucleic acid; **BCH:** Beira Central Hospital; **MCH:** Maputo Central Hospital **NCH:** Nampula Central Hospital; **INS:** Instituto Nacional de Saúde; **IPD:** invasive pneumococcal diseases; **IQR:** interquartile range; **LP:** lumbar puncture; **qPCR:** multiplex qualitative polymerase chain reaction; **NICD**: National Institute for Communicable Diseases; **NRML:** National Reference Microbiology Laboratory; **NTP:** non-typable pneumococci; **NVT:** non-vaccine type; **PCV-10:** ten-valent conjugate vaccine; **PCV-13:** thirteen-valent conjugate vaccine; **RRL:** Regional Reference Laboratory; **SM-PCR NT:** not-typeable by sequential multiplex polymerase chain reaction; **SM-PCR:** sequential multiplex polymerase chain reaction; **UFCSPA:** Universidade Federal de Ciências de Saúde de Porto Alegre.
