# Supplementary material for: Long-Term Impact of Pneumococcal Conjugate Vaccines on the Burden of Pneumococcal Meningitis in Mozambique, 2013–2023
Source: Vaccines (Basel). 2025 Dec 15;13(12):1246. doi: 10.3390/vaccines13121246 (PMC12737670; doi:10.3390/vaccines13121246)
Supplement: Supplementary file 1 [file vaccines-13-01246-s001.zip › vaccines-3594798-supplementary.pdf]

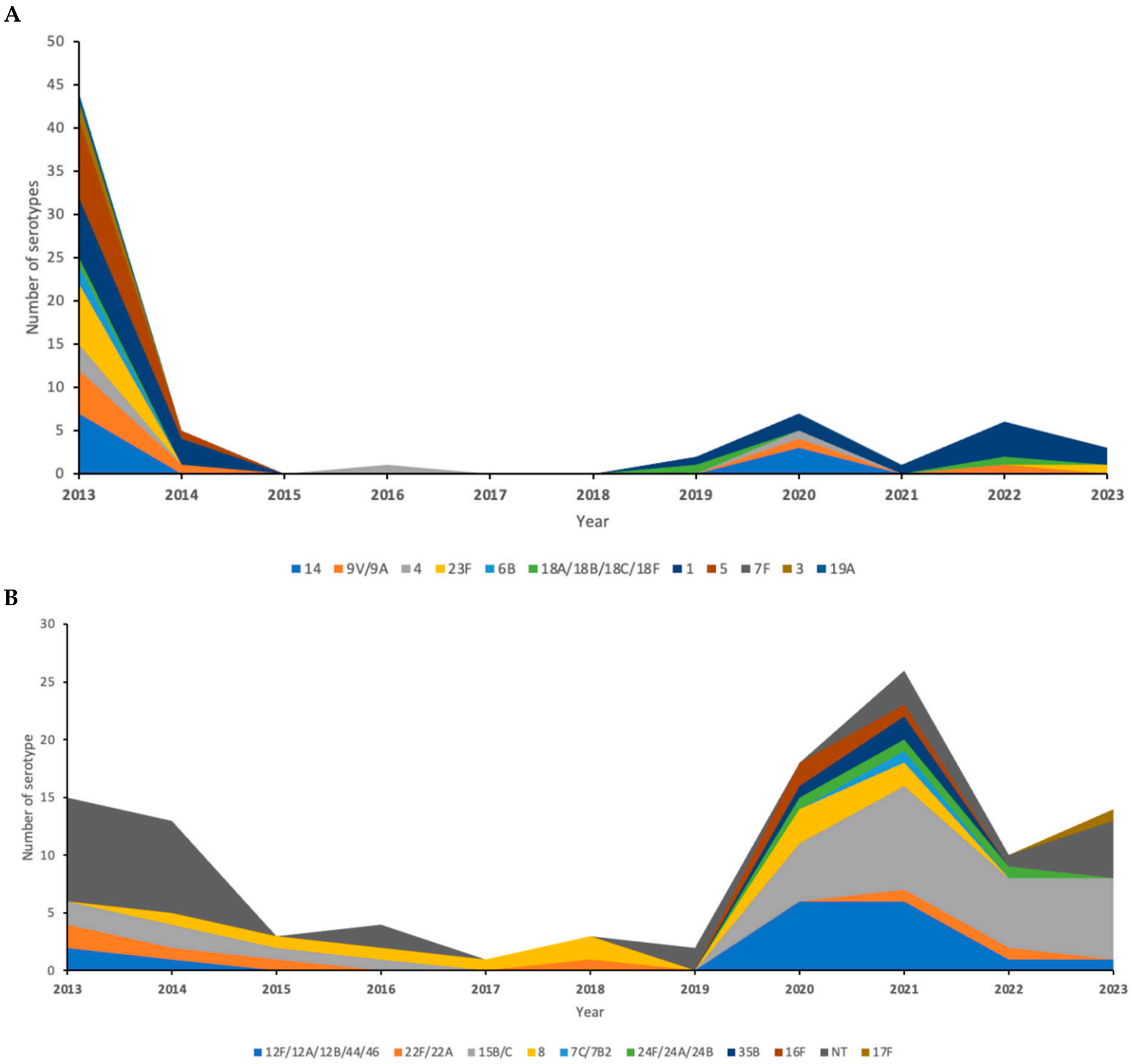

Supplementary Figure S1: Annual counts of serotype-specific *Streptococcus pneumoniae* from pneumococcal meningitis in Mozambique, 2013–2023

(A) PCV13 serotypes in children aged under 5 years old. (B) Non-PCV13 serotypes in children under 5 years old.

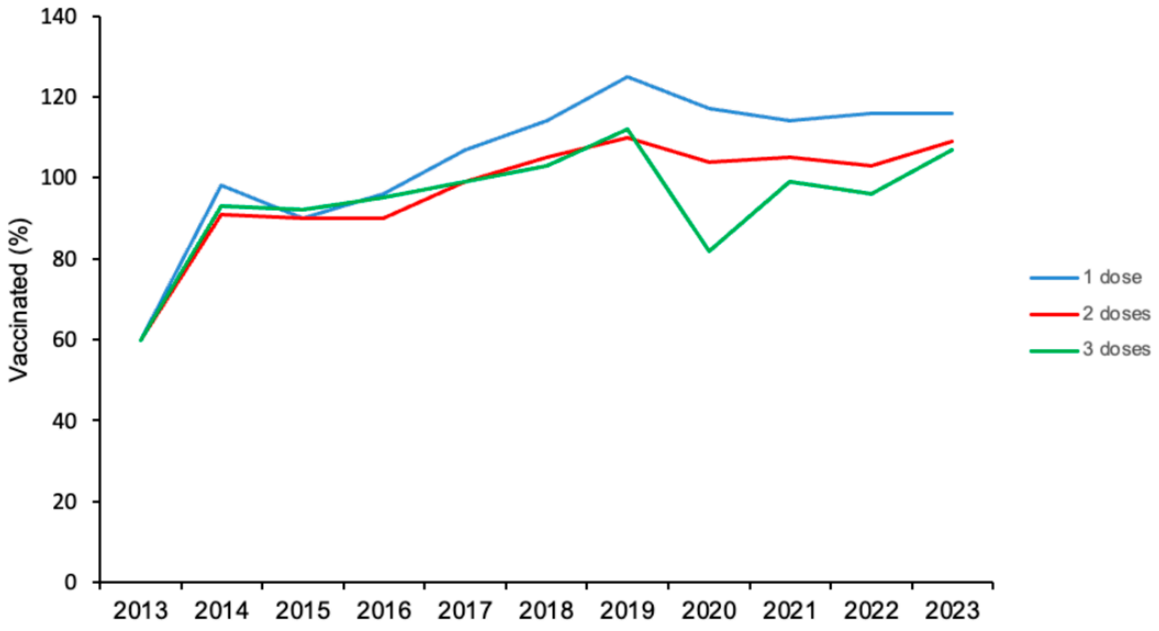

Supplementary Figure S2: Proportion vaccine coverage among children under 1 year old vaccinated with PCV vaccines between 2013 and 2023.

Supplementary Table S1: Catchment population by each hospital per year based on 2007 census (population from 2013-2016) and 2017 census (population from 2017-2023)

**Study Site**	**Age**	**Year**										
		**2013**	**2014**	**2015**	**2016**	**2017**	**2018**	**2019**	**2020**	**2021**	**2022**	**2023**
**Nampula Central Hospital**	<5 Years (Total)	83,232	84,700	86,037	87,231	138,845	136,235	133,949	133,149	135,945	166,864	178,222
	0-11 months	18,949	19,272	19,551	19,784	20,818	28,276	28,960	29,806	30,663	31,560	32,502
	12 a 23 months	31,499	28,788	30,583	29,677	57,425	47,562	47,985	55,890	57,449	59,176	60,954
	24 a 59months	32,784	36,640	35,903	37,770	60,602	60,397	57,004	47,453	47,833	76,128	84,766
**Beira Central Hospital**	**Age**	**Year**										
		**2013**	**2014**	**2015**	**2016**	**2017**	**2018**	**2019**	**2020**	**2021**	**2022**	**2023**
	<5 Years (Total)	57,187	56,318	55,504	54,737	88,115	91,531	95,191	99,578	105,715	129,600	114,651
	0-11 months	12,110	11,967	11,816	11,659	14,853	22,574	22,966	23,570	24,129	24,688	2,014
	12 a 23 months	22,539	22,176	21,844	21,539	36,040	31,847	36,591	44,444	45,465	46,620	68,285
	24 a 59 months	22,539	22,176	21,844	21,539	37,222	37,110	35,634	31,564	36,121	58,292	44,352
**Maputo Central Hospital**	**Age**	**Year**										
		**2013**	**2014**	**2015**	**2016**	**2017**	**2018**	**2019**	**2020**	**2021**	**2022**	**2023**
	<5 Years (Total)	136,989	136,161	135,530	135,097	116,454	114,949	113,837	113,724	115,552	137,801	194,494
	0-11 months	27,449	27,453	27,425	27,366	19,353	26,199	25,888	25,686	25,456	25,209	11,967
	12 a 23 months	53,770	50,354	53,053	52,866	47,261	40,714	43,197	49,645	49,221	48,873	44,352
	24 a 59 months	55,770	58,354	55,053	54,866	49,840	48,036	44,752	38,393	40,875	63,719	138,175

Supplementary Table S2: Pneumococcal meningitis incidence among children under 5 years old, in the PCV10 (3p+0) period (2013-2017) and PCV13 (2p+1) period (2020-2023) in Mozambique

**Characteristic**	**PCV10 (3p+0) period (2013-2017)**			**PCV13 (2p+1) period (2020-2023)**			**PCV13 period vs PCV10 period**	
	Cases (n)	Children at Risk	Incidence per 100,000 (95% CI)	Cases (n)	Children at Risk	Incidence per 100,000 (95% CI)	Incidence rate ratio (95% CI)	*p* -value
**Age (months)**								
0-23	134	849,239	15.8 (13.0–18.5)	73	937,445	7.8 (6.0–9.6)	0.49 (0.4–0.7)	<0.001
24-59	41	602,900	6.8 (4.7-8.9)	30	797,823	3.8 (2.4-5.1)	0.56 (0.3-0.9)	0.012
**Gender**								
Male	98	720,785	13.6 (10.9–16.3)	57	850,610	6.7 (5.0–8.4)	0.49 (0.4-0.7)	<0.001
Female	77	731,354	10.5 (8.2–12.9)	46	884,658	5.2 (3.7–6.7)	0.50 (0.3-0.7)	<0.001

**Sites**								
MCH	49	660,231	7.4 (5.3-9.5)	2	553,793	0.4 (0.1–0.9)	0.05 (0.0–0.2)	<0.001
BCH	5	311,862	1.6 (0.2–3.0)	12	510,154	2.4 (1.0–3.7)	1.50 (0.5-4.2)	0.469
NCH	121	480,046	25.2 (20.7–29.7)	89	671,322	13.3 (10.5–16.0)	0.53 (0.4-0.7)	<0.001
**HIV Status**								
Positive	16	7,544	212.1 (108.2-316.0)	11	18,625	59.1 (24.6–94.0)	0.28 (0.1-0.6)	<0.001
Negative	159	1,444,595	11.0 (9.3- 2.7)	92	1,716,644	5.4 (4.3–6.5)	0.49 (0.4-0.6)	<0.001

Note: **BCH:** Beira Central Hospital; **CI**: confidence interval; **MCH:** Maputo Central Hospital; **NCH:** Nampula Central Hospital; **HIV**: Human Immunodeficiency Virus; **PCV**: Pneumococcal Conjugate Vaccine

Supplementary Table S3: Mortality and case fatality ratio (CFR) among children under 5 years with pneumococcal meningitis during PCV10 period (2013-2017) and PCV13 period (2020 - 2023) in Mozambique

**PCV10 period (2013-2017)**				**PCV13/3p+0 period (2020–2023)**			**PCV13/2p+1 period vs PCV10 period**	
**Cases (n)**		**Children at risk**	**Rate (95% CI)**	**Cases (n)**	**Children at risk**	**Rate (95% CI)**	**Rate Ratio (95% CI)**	***p*-value**
Mortality[a]	32	1,452,139	22.0 (15.5 - 28.5)	9	2,247,756	0.40 (0.02 – 0.48)	0.02 (0.015 - 0.16)	<0.001
Case Fatality Rate[b]	32	175	18.3 (16.9- 24.80)	9	103	8.70 (6.75 – 10.93)	0.48 (0.20 - 0.72)	0.007

Note: **CI**: confidence interval; **PCV**: pneumococcal conjugate vaccine

[a]Mortality were expressed per 100,000 children at risk; [b]Case-fatality rate was expressed as percentages.

S1dataset: Paediatric Bacterial Meningitis in Mozambique database 2013-2023

Sample Code	Sex	Age (month)	YEAR	Local Site	PCR Results	PCV and NonPCV	Status vaccinal	HIV Status	Outcome
[illegible]	[illegible]	[illegible]	[illegible]	[illegible]	[illegible]	[illegible]	[illegible]	[illegible]	[illegible]
